# Disparidades en salud y determinantes legislativos en América Latina: análisis comparativo multivariado de la esperanza de vida, la mortalidad infantil y la inmunización en Chile, Colombia y Honduras (2011-2019)

**DOI:** 10.1016/j.aprim.2025.103428

**Published:** 2026-01-14

**Authors:** Camilo Andrés Estupiñan Ruiz, Cristian Correa-Arrieta, Jeisson Andrés Hincapié-Carvajal

**Affiliations:** a Facultad Enfermería, Universidad El Bosque, Bogotá, Colombia; b Biotecnología y Genética S.A., Universidad Manuela Beltrán, Bogotá, Colombia; c Universidad El Bosque, Bogotá, Colombia

**Keywords:** Indicadores del estado de salud, Mortalidad infantil, Esperanza de vida, América Latina, Determinantes sociales de la salud, Políticas de salud, Health status indicators, Infant mortality, Life expectancy, Latin America, Social determinants of health, Health policy

## Abstract

**Objetivo:**

Comparar la evolución de indicadores sanitarios y su relación con marcos legales y políticas de salud en Chile, Colombia y Honduras entre 2011 y 2019.

**Diseño:**

Estudio ecológico retrospectivo de series temporales, con análisis multivariado y comparativo.

**Emplazamiento:**

Datos secundarios agregados a nivel nacional de la Organización Mundial de la Salud (OMS) y la Organización Panamericana de la Salud (OPS).

**Participantes:**

Población general, reflejada en indicadores de esperanza de vida, mortalidad infantil y cobertura vacunal.

**Intervenciones:**

Revisión de leyes, planes y políticas de salud pública vigentes, incluidas normativas de financiamiento y programas de vacunación.

**Mediciones principales:**

Esperanza de vida al nacer (años), tasa de mortalidad infantil (por 1.000 nacidos vivos), cobertura de vacuna conjugada neumocócica de tres dosis (PCV3, %), marco normativo y políticas de salud.

**Análisis:**

Análisis multivariado de la varianza (MANOVA) para diferencias multivariadas, regresión lineal múltiple ajustada por país y año, correlación de Pearson y diagnóstico de supuestos.

**Resultados:**

Se encontró heterogeneidad significativa entre países (Pillai's trace = 1.0653; p < 0,001). En 2019, Chile mostró mayor esperanza de vida (79,9 años) y menor mortalidad infantil (7,04/1.000), mientras Honduras registró los peores indicadores (71,3 años; 18,8/1.000). La cobertura de PCV3 fue elevada y homogénea (> 90%) en los tres. La correlación entre esperanza de vida y mortalidad infantil fue muy fuerte e inversa (r = –0,943; p < 0,001).

**Conclusiones:**

Las brechas sanitarias responden a interacciones entre factores económicos, institucionales y legislativos. Reducirlas exige fortalecer el marco legal, aumentar el gasto público en salud y promover la cooperación internacional.

## Abstract (en)

**Objective:**

To compare the evolution of health indicators and their relationship with legal frameworks and health policies in Chile, Colombia, and Honduras between 2011 and 2019.

**Design:**

Retrospective ecological study of time series, with multivariate and comparative analysis.

**Setting:**

Secondary national-level data from PAHO and WHO.

**Participants:**

General population, represented through indicators of life expectancy, infant mortality, and vaccination coverage.

**Interventions:**

Review of laws, plans, and public health policies in force, including financing regulations and vaccination programs.

**Main measurements:**

Life expectancy at birth (years), infant mortality rate (per 1,000 live births), coverage of three doses of pneumococcal conjugate vaccine (PCV3, %), legal framework and health policies.

**Analysis:**

MANOVA for multivariate differences, multiple linear regression adjusted by country and year, Pearson correlation, and assumption diagnostics.

**Results:**

Significant heterogeneity was found among countries (Pillai's trace = 1.0653; p < 0.001). In 2019, Chile showed the highest life expectancy (79.9 years) and the lowest infant mortality (7.04/1,000), while Honduras recorded the worst indicators (71.3 years; 18.8/1,000). PCV3 coverage was high and homogeneous (>90%) in all three countries. The correlation between life expectancy and infant mortality was very strong and inverse (r = –0.943; p < 0.001).

**Conclusions:**

Health disparities are explained by interactions among economic, institutional, and legislative factors. Reducing them requires strengthening the legal framework, increasing public health expenditure, and promoting international cooperation.

## Introducción

La medición comparativa de los indicadores básicos de salud constituye un componente esencial para evaluar el desempeño de los sistemas sanitarios y la efectividad de las políticas públicas entre países^1^, ^2^. Entre estos indicadores, la esperanza de vida al nacer, la mortalidad infantil y la cobertura de vacunación reflejan aspectos fundamentales del estado de salud poblacional, estrechamente relacionados con el acceso a los servicios de salud y los determinantes sociales y económicos subyacentes^3^.

América Latina y el Caribe (ALC) exhiben una marcada heterogeneidad estructural, económica y sanitaria, que condiciona el logro de los objetivos de cobertura sanitaria universal^4^. En este contexto, países como Chile, Colombia y Honduras representan trayectorias contrastantes de desarrollo sanitario, financiero e institucional, generando diferencias sustanciales en sus resultados epidemiológicos y en la capacidad de sus sistemas de salud para responder de forma equitativa a las necesidades poblacionales^5^, ^6^. La Organización Mundial de la Salud (OMS) y la Organización Panamericana de la Salud (OPS) han promovido marcos comunes mediante los Objetivos de Desarrollo Sostenible (ODS) y el Programa Ampliado de Inmunización (PAI), con el propósito de reducir las desigualdades sanitarias y fortalecer los sistemas nacionales^7^, ^8^. Sin embargo, la implementación de estos marcos internacionales depende críticamente de la capacidad normativa, de financiamiento y de gobernanza de cada país^9^.

En este contexto, para el presente estudio se tuvo como objetivo comparar tres de los indicadores de salud en Chile, Colombia y Honduras durante el periodo 2011–2019^1^, ^2^. Además, se examinaron las diferencias normativas y de políticas públicas sanitarias que podrían estar modulando las variaciones observadas.

## Metodología

### Diseño del estudio

Se realizó un estudio observacional comparativo y multivariado, de tipo retrospectivo con análisis de series temporales agregadas, utilizando datos secundarios de fuentes oficiales internacionales. El objetivo fue comparar los indicadores básicos de salud entre tres países de ALC (Chile, Colombia y Honduras) durante el periodo 2011-2019, incorporando además un análisis legislativo y de políticas públicas relacionadas.

### Fuentes de datos

Los datos de los indicadores seleccionados fueron obtenidos del repositorio de Indicadores Básicos de Salud de la OPS^1^. Las definiciones y estándares metodológicos internacionales de los indicadores se basaron en las guías del *Global Health Observatory* (GHO) de la OMS^2^. Ambas fuentes proporcionan información estandarizada y validada sobre indicadores demográficos, epidemiológicos y sanitarios a nivel nacional.

### Selección de países e indicadores

Se seleccionaron tres países de América Latina representativos de distintos perfiles socioeconómicos y de desarrollo sanitario, con el fin de explorar la variabilidad en los indicadores básicos de salud regionales: Chile (alto desarrollo sanitario y económico), Colombia (desarrollo intermedio) y Honduras (contexto de mayores desafíos estructurales en salud pública).

Los indicadores de salud analizados incluyeron: esperanza de vida al nacer (expresada en años), tasa de mortalidad infantil (defunciones por 1.000 nacidos vivos), y cobertura de vacunación con la tercera dosis de la vacuna conjugada contra neumococo (PCV3) (porcentaje de niños menores de 1 año que recibieron el esquema completo).

El periodo de análisis abarcó los años 2011 a 2019, correspondiente a la disponibilidad continua y homogénea de datos oficiales para los tres países durante dicho intervalo.

### Variables de estudio

Se definieron como variables dependientes los siguientes indicadores de salud: esperanza de vida al nacer (años), tasa de mortalidad infantil (defunciones por 1.000 nacidos vivos) y cobertura de vacunación con la tercera dosis de la vacuna conjugada contra neumococo (PCV3) (porcentaje de cobertura en menores de un año). Las variables independientes fueron el país (variable categórica) y el año (variable continua).

### Análisis estadístico

Se efectuó inicialmente un análisis descriptivo mediante el cálculo de medidas de tendencia central (media) y dispersión (desviación estándar) para cada indicador y país.

Posteriormente, se aplicó un análisis multivariado de la varianza (MANOVA) para evaluar diferencias globales simultáneas entre los países considerando los tres indicadores de forma conjunta.

Adicionalmente, se ajustaron modelos de regresión lineal múltiple independientes para cada indicador, incluyendo como predictores el país y el año, con el fin de analizar diferencias específicas ajustadas temporalmente y examinar tendencias longitudinales durante el periodo de estudio.

Los supuestos de los modelos (normalidad de residuos, homocedasticidad, linealidad y ausencia de observaciones influyentes) fueron verificados mediante inspección gráfica de residuos y análisis diagnóstico de regresión (Q-Q plots, *Scale-Location, leverage* y distancia de Cook). Se exploraron también las correlaciones entre los indicadores mediante matrices de correlación multivariada y diagramas de dispersión bivariada (*scatterplot* matrices).

El procesamiento y análisis estadístico se realizaron con el software R, versión 4.3.2^10^. Se emplearon los paquetes tidyverse, ggplot2, GGally y reshape2 para la gestión de datos, análisis estadísticos y generación de representaciones gráficas.

Se consideró un nivel de significación estadística de p < 0,05 en todos los análisis.

### Consideraciones éticas

Este estudio se desarrolló exclusivamente mediante el análisis de bases de datos públicas, desagregadas por país y anónimas, disponibles en los repositorios oficiales de la OMS y de la OPS. Por tanto, no fue requerida la aprobación por parte de un comité de ética en investigación.

## Resultados

**Figura 1:**
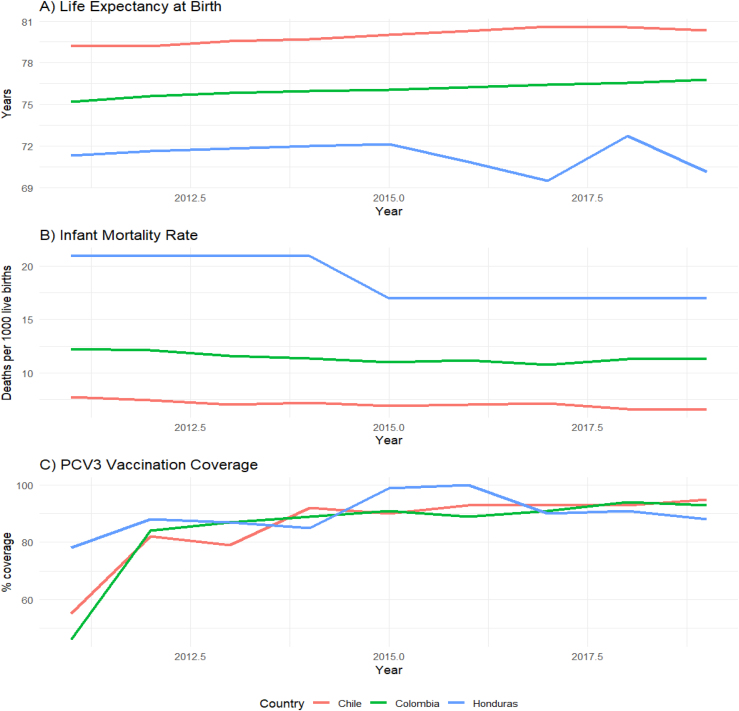
Tendencia temporal de los indicadores básicos de salud en Chile, Colombia y Honduras (2011-2019). A) Esperanza de vida al nacer (en años); B) Tasa de mortalidad infantil (defunciones por 1.000 nacidos vivos); C) Cobertura de vacunación con la tercera dosis de vacuna conjugada contra neumococo (PCV3) (%). Se observa un patrón consistente de mejores resultados en Chile para la esperanza de vida y la mortalidad infantil, con diferencias intermedias en Colombia y mayores rezagos en Honduras. La cobertura vacunal muestra niveles altos y tendencias ascendentes en los tres países, con variaciones interanuales. Los datos corresponden a registros oficiales provenientes de OPS y OMS.

El análisis descriptivo evidenció diferencias sistemáticas entre los países en los tres indicadores de salud analizados (tabla 1, fig. 1). La esperanza de vida al nacer mostró un gradiente marcado, con Chile en la posición más favorable (79,9 años), seguido de Colombia (76,1) y Honduras (71,3). En la mortalidad infantil, Chile también presentó la tasa más baja (7,04 por 1.000 nacidos vivos), mientras que Colombia y Honduras registraron cifras mayores (11,4 y 18,8, respectivamente). En contraste, la cobertura de vacunación con tres dosis de la vacuna conjugada neumocócica (PCV3) fue elevada en los tres países, con valores medios superiores al 84%. Honduras alcanzó la cobertura más alta (89,6%), seguida de Chile (85,8%) y Colombia (84,9%).

**Tabla 1:** Evolución de los indicadores de salud en Chile, Colombia y Honduras (2011-2019)

País	Año	Esperanza de vida (años)	Mortalidad infantil (por 1.000 NV)	Cobertura PCV3 (%)
Chile	2011	79,24	7,7	55
	2012	79,22	7,4	82
	2013	79,55	7,0	79
	2014	79,71	7,2	92
	2015	80,01	6,9	90
	2016	80,30	7,0	93
	2017	80,61	7,1	93
	2018	80,56	6,6	93
	2019	80,32	6,5	95
Colombia	2011	75,18	12,25	46
	2012	75,61	12,14	84
	2013	75,83	11,56	87
	2014	75,95	11,34	89
	2015	76,07	10,96	91
	2016	76,23	11,15	89
	2017	76,42	10,73	91
	2018	76,58	11,29	94
	2019	76,79	11,35	93
Honduras	2011	71,32	21,0	78
	2012	71,62	21,0	88
	2013	71,81	21,0	87
	2014	72,00	21,0	85
	2015	72,12	17,0	99
	2016	70,86	17,0	100
	2017	69,49	17,0	90
	2018	72,71	17,0	91
	2019	70,12	17,0	88

**Figura 2:**
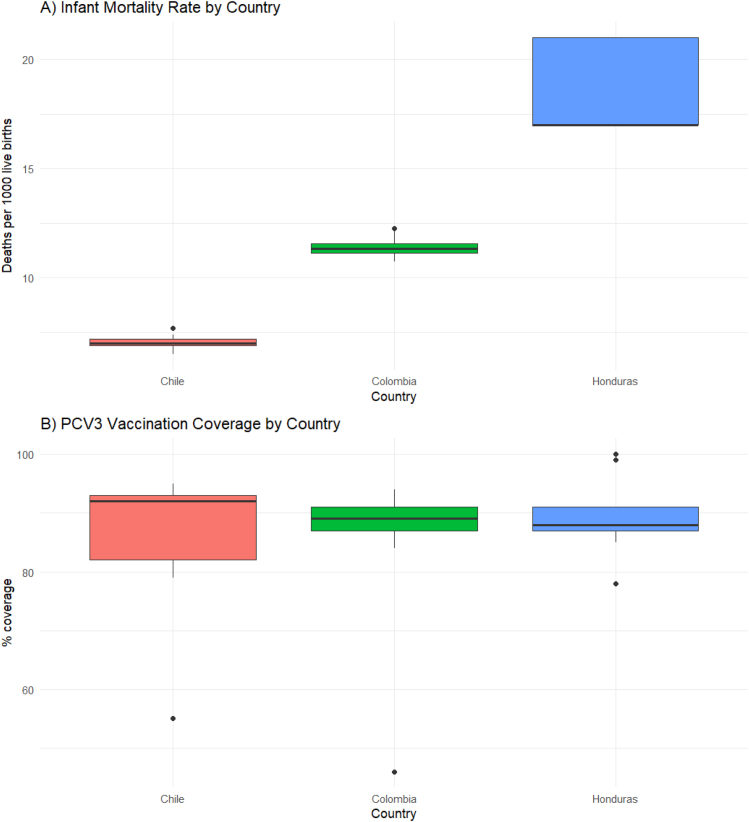
Distribución de la mortalidad infantil y cobertura de vacunación PCV3 en Chile, Colombia y Honduras (2011-2019). A) Distribución de la tasa de mortalidad infantil (defunciones por 1.000 nacidos vivos) por país. B) Distribución de la cobertura de vacunación con tercera dosis de vacuna conjugada contra neumococo (PCV3) (%). Los diagramas de caja ilustran las diferencias interpaís en los niveles centrales y la dispersión de cada indicador durante el periodo de estudio. Se observan mayores tasas de mortalidad infantil en Honduras, diferencias intermedias en Colombia y los mejores resultados en Chile. La cobertura vacunal muestra alta mediana en los tres países, con mayor dispersión en Honduras y valores atípicos ocasionales.

El análisis multivariado mediante MANOVA confirmó la existencia de diferencias estadísticamente significativas en los indicadores de salud entre los tres países (Pillai's trace = 1.0653; F = 8.7379; gl = 6/46; p < 0,001). La prueba de Wilks corroboró este resultado (λ=0,0108; F = 63.379; gl = 6/44; p < 0,001), indicando que más del 98% de la varianza conjunta se atribuye a las diferencias entre países (fig. 2).

Los análisis univariados permitieron precisar estas diferencias. Para la esperanza de vida al nacer, el modelo de regresión múltiple (R^2^ ajustado = 96,3%) mostró que el país era el factor determinante. Con Chile como referencia, Colombia presentó una reducción promedio de 3,87 años (p < 0,001), mientras que Honduras alcanzó una diferencia de 8,60 años (p < 0,001). La variable año no resultó significativa (p = 0,138), sugiriendo estabilidad temporal en el periodo 2011–2019 (fig. 1).

En la mortalidad infantil, el modelo ajustado (R^2^ ajustado = 96,3%) mostró incrementos significativos en comparación con Chile: + 4,37 defunciones/1.000 nacidos vivos en Colombia y + 11,73 en Honduras (ambos p < 0,001). A diferencia de la esperanza de vida, aquí sí se identificó una tendencia decreciente en el tiempo (–0,307 defunciones/1.000 por año; p < 0,001) (fig. 1).

**Figura 3:**
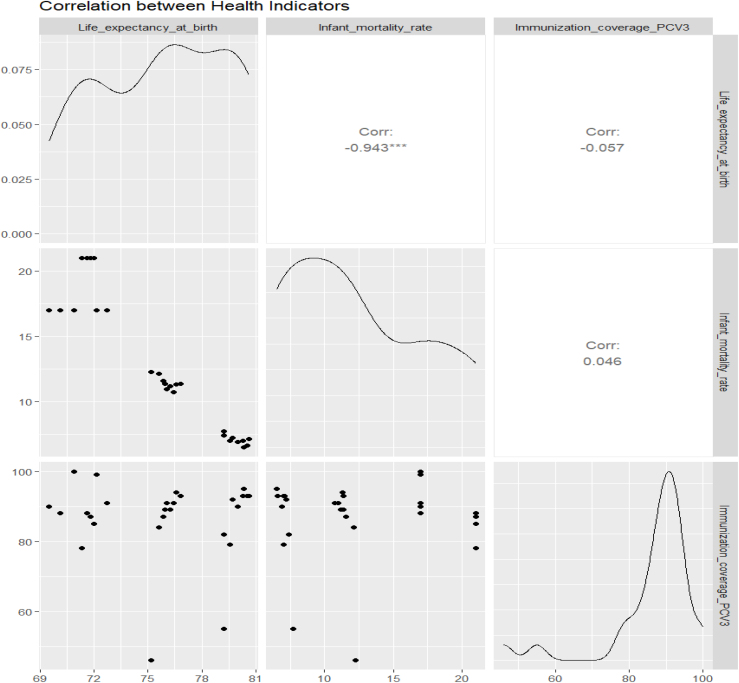
Matriz de correlación entre los indicadores de salud analizados (2011-2019). Matriz de dispersión y correlación entre esperanza de vida al nacer (años), tasa de mortalidad infantil (defunciones por 1.000 nacidos vivos) y cobertura de vacunación PCV3 (%). Se observa una fuerte correlación inversa entre esperanza de vida al nacer y mortalidad infantil (r = -0,943; p < 0,001), mientras que no se identificaron correlaciones estadísticamente significativas entre la cobertura vacunal y los otros dos indicadores. Las diagonales muestran las distribuciones de densidad de cada variable. Los gráficos inferiores representan los diagramas de dispersión bivariados para cada combinación de variables.

En cuanto a la cobertura de vacunación, el modelo explicó menor variabilidad (R^2^ ajustado = 37,8%). No se observaron diferencias significativas entre países (p > 0,05), pero sí un aumento temporal sostenido (+2,88% anual; p < 0,001), lo que refleja un proceso de mejora continua en la inmunización infantil (Figura 2, Figura 3).

El análisis de correlación evidenció una asociación inversa y robusta entre esperanza de vida al nacer y mortalidad infantil (r = –0,943; p < 0,001). No se encontraron asociaciones significativas entre la cobertura de PCV3 y los otros indicadores, lo que sugiere independencia estadística (fig. 2).

Finalmente, los diagnósticos de los modelos confirmaron un buen ajuste, sin violaciones relevantes de los supuestos. Los residuos se distribuyeron de manera aleatoria y cercana a la normalidad. Solo en el modelo de cobertura vacunal se detectó ligera heterocedasticidad, sin comprometer la validez de los resultados (fig. 3).

## Discusión

Los hallazgos confirman la existencia de desigualdades significativas en los indicadores básicos de salud entre Chile, Colombia y Honduras durante 2011–2019. La evidencia multivariada y univariada indica que el país de residencia es un determinante estructural en la configuración de la esperanza de vida, la mortalidad infantil y, en menor medida, la cobertura vacunal.

La correlación inversa encontrada entre esperanza de vida y mortalidad infantil refuerza la validez de ambos indicadores como reflejo del estado de salud poblacional. La estabilidad de la cobertura vacunal, elevada y homogénea destaca el éxito de los programas de inmunización infantil en América Latina bajo los lineamientos de la OPS, a pesar de las diferencias socioeconómicas y estructurales entre países.

### Diferencias inter-país y sistemas sanitarios

Chile mostró consistentemente los mejores indicadores, lo que se asocia a un sistema mixto de financiamiento (FONASA e ISAPRE) y al impacto del Plan AUGE (Ley 19.966), que garantiza acceso equitativo a servicios esenciales. La institucionalización de las garantías explícitas en salud (GES) ha favorecido la equidad y la protección financiera de los hogares^11^.

Colombia, con la Ley 100 de 1993 y la Ley Estatutaria 1751 de 2015^12^, ^13^, logró avances notables en aseguramiento universal, pero persisten desigualdades territoriales y sociales^14^, ^15^. Poblaciones rurales, indígenas y desplazadas siguen presentando barreras de acceso que afectan negativamente los indicadores^15^, ^16^, ^17^.

Honduras, en cambio, enfrenta limitaciones estructurales más severas: altos niveles de pobreza, bajo gasto público en salud, fragmentación institucional y déficit de infraestructura. Todo ello se traduce en los indicadores más desfavorables, especialmente en la mortalidad infantil. La cooperación internacional, en particular a través de GAVI y la OPS, ha contribuido a sostener coberturas vacunales altas, pero no ha logrado compensar las brechas en otros determinantes estructurales.

### Determinantes sociales de la salud

Las desigualdades entre países reflejan la influencia de determinantes sociales como pobreza, desigualdad de ingresos, nivel educativo y acceso desigual a servicios básicos. La Comisión de Determinantes Sociales de la OMS ha señalado que estas inequidades son evitables y responden a políticas económicas y sociales insuficientes^18^, ^19^.

Honduras concentra los mayores desafíos, con índices altos de pobreza multidimensional y barreras de acceso a servicios de salud. Colombia ha avanzado en cobertura, pero mantiene brechas profundas en áreas rurales y poblaciones vulnerables^20^. Chile ha implementado políticas intersectoriales que han contribuido a mejorar condiciones educativas, económicas y sanitarias, lo que se traduce en mejores resultados poblacionales^21^.

### Marcos normativos y regulatorios

Los marcos legales nacionales son determinantes clave. En Chile, el GES/AUGE ha consolidado la atención priorizada y el acceso financiero protegido. En Colombia, la salud se reconoce como derecho fundamental, lo que ha ampliado el acceso. Honduras promulgó en 1998 la Ley Marco del Sistema Nacional de Salud, pero su implementación ha sido limitada por la insuficiencia presupuestaria y la debilidad institucional^22^.

A nivel regional, los tres países han mantenido sus PAI activos bajo lineamientos de la OPS, lo que ha permitido preservar coberturas elevadas y homogéneas^23^, ^24^.

### Contexto internacional y cooperación

La Agenda 2030 y los ODS, en particular el ODS 3, han impulsado compromisos globales en salud. El PAI, junto con el apoyo financiero de GAVI^25^, ha sido decisivo para garantizar la vacunación infantil en países de ingresos bajos como Honduras. La OPS y la OMS han liderado estrategias regionales que integran políticas basadas en evidencia, atención materno-infantil y sistemas de vigilancia sanitaria^26^.

### Limitaciones

Este estudio presenta limitaciones metodológicas: uso de fuentes secundarias agregadas, inclusión de solo tres países, periodo relativamente corto (2011–2019) y ausencia de variables micro (conductas individuales, factores culturales). Tampoco se exploraron en detalle aspectos políticos o de gobernanza que influyen en los resultados sanitarios.

## Conclusión

El presente análisis multivariado comparativo evidencia la existencia de desigualdades persistentes en los indicadores básicos de salud entre Chile, Colombia y Honduras durante el periodo 2011-2019, reflejando el impacto combinado de factores estructurales, socioeconómicos, institucionales y legislativos sobre el desempeño sanitario de cada país. Si bien los programas de inmunización muestran resultados homogéneos a nivel regional, persisten brechas significativas en esperanza de vida y mortalidad infantil, particularmente en contextos de mayores limitaciones económicas e institucionales. Estos hallazgos destacan la necesidad de fortalecer los marcos normativos nacionales, avanzar en la universalización efectiva de la cobertura sanitaria, priorizar la atención materno-infantil, y consolidar la gobernanza sanitaria a largo plazo. La acción intersectorial coordinada, el fortalecimiento de capacidades nacionales y el apoyo sostenido de la cooperación internacional continúan siendo elementos estratégicos indispensables para reducir las inequidades sanitarias en América Latina y alcanzar los compromisos establecidos en los ODS.

## Autoría y uso de IA

Este trabajo fue elaborado por los autores mencionados. No se utilizó inteligencia artificial para la generación de contenido sustantivo. Los autores declaran haber participado de manera sustancial en todas las etapas del trabajo: en la concepción y diseño del estudio, la adquisición, análisis e interpretación de los datos; en la redacción del manuscrito y la revisión crítica de su contenido intelectual; y en la aprobación final de la versión presentada para su publicación.

## Declaración de transparencia

El manuscrito presentado es honesto, preciso y transparente en todos sus aspectos; no se han omitido datos relevantes y las discrepancias con las fuentes originales han sido explicadas

## Financiación

Los autores declaran que para la presente investigación ha contado con el aval y el apoyo de la Universidad El Bosque, con descargas de hora.

## Consideraciones éticas

Este estudio se basa únicamente en datos secundarios agregados de dominio público (PAHO/WHO), por lo que no requirió aprobación de un comité de ética ni la obtención de consentimiento informado.

## Conflicto de intereses

Los autores declaran no tener ningún conflicto de intereses en relación con el presente trabajo.

## Agradecimiento

Al grupo de investigación de Cuidado de la salud y calidad de Vida adscrito a la Facultad de Enfermería de la Universidad El Bosque.
